# Person-centered pain management – science and art

**DOI:** 10.3325/cmj.2013.54.296

**Published:** 2013-06

**Authors:** Marijana Braš, Veljko Đorđević, Mladen Janjanin

**Affiliations:** 1 Centre for Palliative Medicine, Medical Ethics and Communication Skills, University of Zagreb School of Medicine, Zagreb, Croatia *mbras@kbc-zagreb.hr*; 2 Academy of Music, University of Zagreb, Zagreb, Croatia

## Abstract

We are witnessing an unprecedented development of the medical science, which promises to revolutionize health care and improve patients’ health outcomes. However, the core of the medical profession has always been and will be the relationship between the doctor and the patient, and communication is the most widely used clinical skill in medical practice. When we talk about different forms of communication in medicine, we must never forget the importance of communication through art. Although one of the simplest, art is the most effective way to approach the patient and produce the effect that no other means of communication can achieve. Person-centered pain management takes into account psychological, physical, social, and spiritual aspects of health and disease. Art should be used as a therapeutic technique for people who suffer from pain, as well as a means of raising public awareness of this problem. Art can also be one of the best forms of educating medical professionals and others involved in treatment and decision-making on pain.

Today, we are witnessing unprecedented advances in medical science that promise to significantly improve patients’ health outcomes. Personalized medicine is the latest life sciences innovation, which uses each person’s unique clinical, genetic, genomic, and environmental information. However, the core of the medical profession has always been and will be the relationship between the doctor and the patient (1). Due to recent advances in neuroscience, we are now able to describe and discuss the neurobiology of doctor-patient relationship (2). Different physiological and biochemical mechanisms take part in complex functions, like trust, hope, empathy, and compassion, which are all very important elements in the doctor-patient relationship. From neuroscientific perspective, the doctor-patient relationship can be divided into at least four steps: feeling sick, seeking relief, meeting the therapist, and receiving therapy (3). With the help of neuroscience, health professionals can better understand the changes they induce in their patients' brains (3). Modern physicians need to be educated on how to use their highly specialized knowledge when approaching an individual patient as a unique and whole person living in a given psychological, social, and material context. Such an approach combines the best of ancient medicine – which was individualized and person-oriented, yet not sufficiently scientific – and modern, scientific-oriented, highly efficient medicine, which, unfortunately, tends to disregard the person as a whole (1). The earliest roots of person-oriented medicine may be found in ancient civilizations, which emphasized a holistic approach to the person, health, and disease. This concept is expressed in the definition of health (18), as well as in the personalized approach to health care. Person-centered medicine is complementary to personalized medicine, and takes into account the psychological, physical, social, and spiritual aspects of health and disease. Person-centered medicine observes both the patient and the health care professional as people in a cooperative partnership, emphasizing totality and wholeness of each person. Person-centered medicine is committed to equal opportunities for all, especially to the availability of care, with an emphasis on autonomy and individual’s rights to health care (4,5).

## The art of communication skills in person-centered medicine

As health care in general is a very complex system, reflecting social changes, in recent decades great attention has been paid to the quality of communication in medicine. Communication is the most widely used clinical skill in medical practice, which we use for hundreds of thousands of times in our working lives. It includes a large number of interactions with all participants in the health system. Training in communication skills in medicine is not just an event, but a process, because it is a clinical skill that is essential for a long-term theoretical, practical, individual, and team work (6). The paternalistic relationship, where the doctor is omniscient and makes all decisions related to patient's health, is slowly becoming a matter of the past. According to the today’s model of collaborative partnership, communication between patients and doctors should be based on a common understanding in a caring and dynamic relationship, which must also involve the patient’s family (7). In other words, it is a process in which the communication with the patients, their families, and within the health care team is a precondition for everyone’s success (8). The medical interview provides a framework for exploring and understanding patients' concerns, fears, and misconceptions, while taking into consideration their culture, availability of treatment options, and financial considerations. Person-centered medical interview is an important bridge between personalized and person-centered medicine (9).

When we talk about different forms of communication in medicine, we must never forget the importance of communication through art. Patients express their emotions toward their therapists by means of pictures, paintings, drama, or music. Although one of the simplest, art is the most effective way to approach the patient and produce the effect that no other means of communication can achieve. Art, as a universal language, is maybe the most powerful means to connect and improve people from all over the world. Art influences people's emotions and forms an invaluable part of today's medicine (1). Creation is an act of survival, growth, and development of an individual and the community. There is no development without creativity.

Since medicine is creative, the doctor is a creator too. Doctors do not have a canvas, do not write music, their work is the result of the relationship with a person or a couple, family or a group they work with. Person-centered medicine, characterized by an encounter of two personalities who solve problems together, is a new and irreproducible creation, because it uses past experiences and acquired knowledge to create a new quality. This process differs across cultures, but the mechanism is always the same or similar, the creation is individual and the experience unique. When talking about “The Care of the Patient,” in a series of talks before students of the Harvard Medical School, Francis W. Peabody said: “The practice of medicine in its broadest sense includes the whole relationship of the physician with his patient. It is an art, based to an increasing extent on the medical sciences, but comprising much that still remains outside the realm of any science. The treatment of a disease may be entirely impersonal; the care of a patient must be completely personal. What is spoken of as a “clinical picture” is not just a photograph of a man sick in bed; it is an impressionistic painting of the patient surrounded by his home, his work, his relations, his friends, his joys, sorrows, hopes and fears. One of the essential qualities of the clinician is interest in humanity, for the secret of the care of the patient is in caring for the patient” (10). A doctor’s approach is always unique and reflects us, as a person, and the context in which the meeting takes place. Each meeting is a new creation, a creation of a relationship that can be recorded or imitated, but never repeated in exactly the same way. That is why medicine and art are connected.

## Art in person-centered pain management

**Figure 1:**
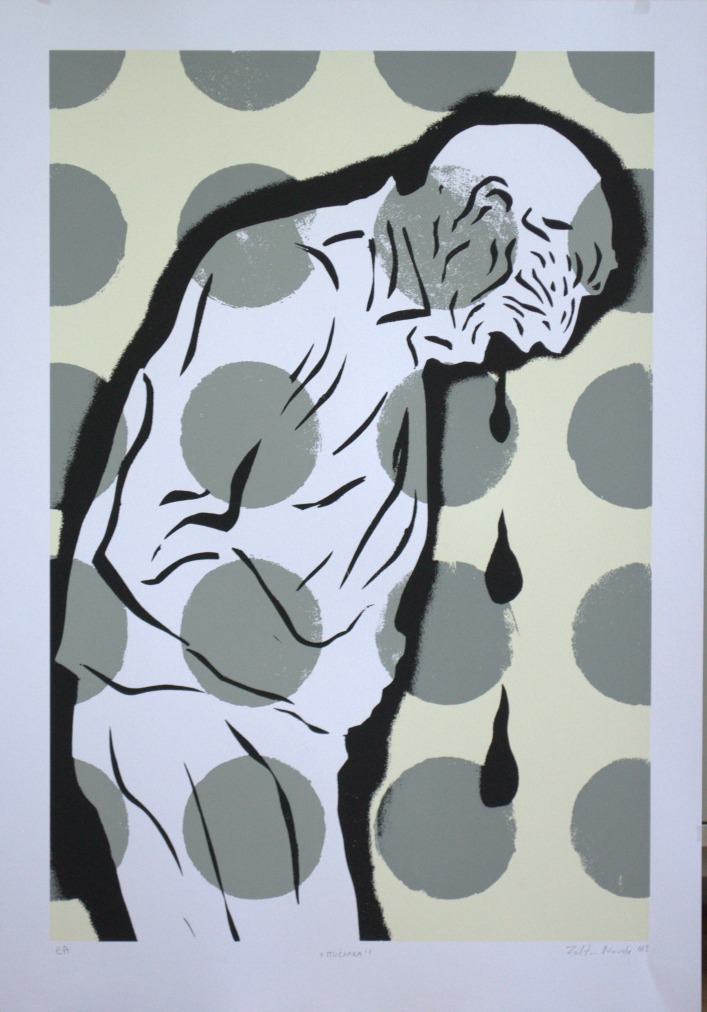
*Nausea*, a drawing by Prof. Zoltan Novak, from the series of artworks by 12 artists inspired by patients’ stories on pain; corresponding story in Box 1

Pain is the most common reason why people consult a doctor, but also the most common symptom indicating the onset of an illness (Box 1) (Figure 1). Pain is the oldest evolutionary reaction of an organism to any kind of attack or destruction. Is pain a signal, a symptom, an emotion or an illness? Why do we feel pain? The cause can be physical, psychological, social, or spiritual. Today, we are witnessing many innovations in prevention, diagnosis, treatment, and rehabilitation of patients with chronic pain, and it is not easy to imagine what the future diagnostic and therapeutic advances will bring (11). Pain is a dominant public health problem in the world, which requires a multidisciplinary approach. Modern science and clinical practice already enable us to significantly reduce pain and suffering regardless of the cause and kind of pain. Pain is a subjective feeling and it is not easy to measure it. Since no two people are the same, no two reactions to pain are the same. It is difficult, almost impossible to compare biologically caused pain with emotional, social, or spiritual pain, but all of them can cause great suffering.

Box 1 **A story by Ž.N., a patient with depression and neuropathic pain in the shoulder; corresponding drawing in Figure 1**

The night is passing slowly and my shoulder has gone mad after the exercises with you. I felt at one moment as if somebody stuck a needle into my eye! I can’t understand where this pain comes from. I should be hit in the shoulder; it reacts if the right place is pressed. I don’t know if I will be able to endure this torture in the future because the man inside me is not me! The old me was unstoppable in my campaigns and on my journeys. I was healthy, responsible, and disciplined, a man with a goal.

The new me – he is nothing, an empty shell who can barely concentrate during our sessions and after that he has to have a rest. I don’t know if I am depressed. I don’t know... I can only see that I am stuck and that I can’t get back into normal life because I lack the strength. I have built a wall around me and the only door in it is opened only for the people I love – there is no place for others.

I am not interested in the outer world. I go out, of course, and I pretend all the time thinking “I can hardly wait to get back home.” I have cared least about myself in these years full of pain, but I care about the shoulder which prevents me from doing most things I want to do. I don’t believe it, it is not my friend, because it lets me down when it mustn’t and shouldn’t. I have easily managed to get rid of such friendships, but I can’t get rid of this one. I am nervous, I don’t have any ideas, I am gloomy, except for the light created by my children. When they are around me I spare them as much I as can.

Sometimes I think that pain leads to madness and I wouldn’t like that.

Maybe I am trapped? Or being tested? I don’t understand anything any more. The physical pain is eating my soul and my body.

Pain – I feel it whenever I touch my body. See, it hurts here. I am sick of pain. It is like when you press something trying to go through it, but it is not possible. It stings. My shoulder muscles have partly atrophied and I feel like I have a temperature. It is as if my shoulder is covered by a sick, slimy, greenish-yellowish liquid, like something not from this planet. It feels like my shoulder is inside a slimy structure causing pain. The pain has made me feel disgusted by my shoulder. I don’t love it any more, because it hurts. The pain has changed my habits, my life, my way of thinking. My life has turned upside-down since it started and the shoulder has become the center of my existence. My standards have dropped dramatically and I have lost my creativity. I feel pity for myself – how long is it going to last? I am angry. I talk to my shoulder, “I have done everything for you.” Maybe it was the doctor’s fault. I don’t sleep well, three hours on average. The rest of the time I have all sorts of thoughts and different reactions. Lethargy, apathy, and antisocial behavior are always present. It seems that my problem can be seen on my face. Which colors can you use to describe pain which goes all the way to the bones? I feel most secure at home, which I love and which somehow understands all that horror I live in and from which I am seeking a way out. I am fed up with myself!

How do we perceive an individual who is seeking medical help because of chronic pain? Do we treat a disease or a person? Pain impairs the functioning of the person in various ways – how much do we see and recognize? To what extent do we understand the patient’s perspective? How do we build the relationship? And to what extent do we manage to see ourselves, our reactions, and problems? What are the best ways to observe a patient with chronic pain as a person within the context of his or her life and environment? It is important to focus on stimulating healthy and creative forces in our patients with chronic pain, which are so important for their coping with disease, maintaining hope, fighting for life, and not giving up. An effort must be made to provide patients with the best possible health care as well with empathy. Today, it is more than ever important to balance humanism and medical sciences in pain management. We should accept the person seeking help as a whole, but that person should also accept us as a person who provides help and can be trusted. Therefore, good communication skills and person-centered medical interview is very important in pain management.

There is a strong and unbreakable bond between pain and art. Pain can motivate us, warn us, and initiate a new way of expressing ourselves though a painting, composition, novel, or a poem. Many works of art have resulted from pain, which should be respected, but it also shows us that art could be used as a therapeutic technique for people who suffer from pain, as well as a means to raise public awareness of this problem.

Why art-therapy in pain management? For over fifty years, creative art therapists have used it in hospital environments to facilitate relaxation, decrease anxiety, and provide distraction. Art therapy is a mental health profession that uses the creative process of art making to improve the physical, mental, and emotional well-being of individuals. Art therapy has grown into an effective and important method of communication, assessment, and treatment of children and adults in a variety of settings, including pain management. Art therapy integrates visual art (drawing, painting, sculpture, and other art forms) and creative process with counseling and psychotherapy. Art therapists are professionals trained in both art and therapy. They use art in treatment, assessment and research, and provide consultations to other professionals.

Our brains function holistically. The left and right hemisphere of the brain manipulate information differently, but the contribution of the right hemisphere to our cultural achievements has only recently become clearer. Creativity can be seen as a basic neurobiological force, which in *Homo sapiens* has evolved to the drive for exploring other worlds (12). Numerous studies show how creative expression through music, writing, or art work can break the cycle of chronic pain. The idea behind this type of therapy is that creative activities facilitate the healing process and rehabilitation (13,14). When communicating with a work of art, we communicate with ourselves and with our past, but also with our future, which is at that moment created through imagination. Our brain is the master of universe in this process – we can travel through time, fantasies, and reality. We can separate ourselves from pain or any other unpleasant feeling. Evolutionary speaking, the invention of rhythm and music precedes that of speech. With rhythm and music we can also express our feelings and needs, often more effectively than with words. Music therapy in particular has a long tradition in the treatment of pain and health disorders. In addition to music therapy, creative arts, creative dance, and movement-based creative therapy have also been used in treatment of patients with chronic pain. They have been show to offer various benefits: enhancing the activity level and creative capacity as a healing source; stimulating positive emotional experience; experiencing social communication and interaction; facilitating projective coping; stimulating imaginative experience and awareness; and promoting suggestive elements (15,16).

Art can also be one of the best forms of educating medical professionals and other people involved in treatment and decision-making on pain. Three years ago, we founded the Center for Palliative Medicine, Medical Ethics, and Communication Skills at the Zagreb University School of Medicine (17). A special point in our development was the Memorandum of Understanding with the Academy of Music, Academy of Dramatic Art and Academy of Fine Arts at the University of Zagreb at the beginning of 2012. This was the key piece of the puzzle, which joined our ideas into a meaningful whole by emphasizing the interrelatedness of science, clinical practice, and art, and the need for understanding the patient and health professional as persons. Together we decided to start the “Communication against Pain” project with an aim to promote the importance of pain management.

## Conclusion

Technological superiority must not make us lose sight of patients as persons in their physical, psychological, social, and spiritual totality. Pain management is a good example of the importance of person-centered medicine. The experience of pain presents a complex interaction of neurological, emotional, cognitive, social, and cultural factors. The model of chronic pain as a phenomenon of consciousness, which depends on subjective perception, communication, and coping is the basis for understanding art therapy in pain management. Art should be used as a therapeutic technique for people who suffer from pain, as well as for raising public awareness of this problem. *Ars medica* (the art of medicine) is both art in medicine and medicine of art, as well as a journey from the culture of illness toward the culture of health, from symptoms and diagnoses toward human beings and persons.
